# The Sensitivity to Pain Traumatization Scale–Child Version (SPTS-C): Development and preliminary validation

**DOI:** 10.1080/24740527.2023.2298769

**Published:** 2024-01-12

**Authors:** Maria Pavlova, Jaimie K. Beveridge, Sabine Soltani, Larah Maunder, Tim V. Salomons, Joel Katz, Melanie Noel

**Affiliations:** a Department of Psychology, University of Calgary, Calgary, Alberta, Canada; b Department of Psychology, Queen’s University, Kingston, Ontario, Canada; c Department of Psychology, York University, Toronto, Ontario, Canada; d Alberta Children’s Hospital Research Institute, Calgary, Alberta, Canada; e Hotchkiss Brain Institute, Calgary, Alberta, Canada; f Mathison Centre for Mental Health Research and Education, Calgary, Alberta, Canada

**Keywords:** pediatric chronic pain, sensitivity to pain traumatization, scale development, factor analysis, dominance analysis

## Abstract

**Background:**

Sensitivity to pain traumatization is defined as the propensity to develop cognitive, affective, and behavioral responses to pain that resemble a traumatic stress reaction. To date, sensitivity to pain traumatization has been assessed in adults (Sensitivity to Pain Traumatization Scale [SPTS-12]) and parents of youth with chronic pain (Sensitivity to Pain Traumatization Scale–Parent version [SPTS-P]). SPT may be relevant in the context of pediatric chronic pain given the substantial comorbidity between posttraumatic stress symptoms and pain.

**Aims:**

This prospective study aimed to adapt the SPTS-12 for use in youth and to evaluate the psychometric properties of the new scale.

**Methods:**

Participants included 175 youth with chronic pain (*M*_age_ = 14.31 years, 73% girls) referred to outpatient chronic pain programs. At baseline, youth self-reported the levels of their sensitivity to pain traumatization (Sensitivity to Pain Traumatization Scale–Child version [SPTS-C]), as well as their pain symptoms, pain-related anxiety, posttraumatic stress symptoms, and attentional control. Three months later, youth self-reported their pain symptoms and completed the SPTS-C.

**Results:**

The SPTS-C had a one-factor structure that explained 48% of variance and demonstrated good reliability and construct validity. SPTS-C baseline scores predicted follow-up levels of pain interference but not pain intensity or pain unpleasantness.

**Conclusions:**

The results provide preliminary evidence for the psychometric properties of the SPTS-C and the potential role of SPT in pediatric chronic pain outcomes.

## RÉSUMÉ

**Contexte:** La sensibilité à la traumatisation de la douleur est définie comme la propension à développer des réponses cognitives, affectives et comportementales à la douleur qui ressemblent à une réaction de stress traumatique. À ce jour, la sensibilité à la traumatisation de la douleur a été évaluée chez les adultes (Échelle de sensibilité à la traumatisation de la douleur [SPTS-12]) et chez les parents de jeunes souffrant de douleur chronique (Échelle de sensibilité à la traumatisation de la douleur - Version parent [SPTS-P]). La sensibilité à la traumatisation de la douleur peut être pertinente dans le contexte de la douleur chronique pédiatrique étant donné la comorbidité importante entre les symptômes de stress post-traumatique et la douleur.

**Objectifs:** Cette étude prospective visait à adapter le SPTS-12 pour une utilisation chez les jeunes et à évaluer les propriétés psychométriques de la nouvelle échelle.

**Méthodes:** Les participants comprenaient 175 jeunes souffrant de douleur chronique (âge M = 14,31 ans, 73 % de filles) référés aux programmes de traitement ambulatoire de la douleur chronique. Au départ, les jeunes ont autodéclaré les niveaux de leur sensibilité à la traumatisation de la douleur (Échelle de sensibilité à la traumatisation de la douleur - version enfant [SPTS-C]), ainsi que leurs symptômes de douleur, leur anxiété liée à la douleur, leurs symptômes de stress post-traumatique et leur contrôle attentionnel. Trois mois plus tard, les jeunes ont autodéclaré leurs symptômes de douleur et ont répondu au SPTS-C.

**Résultats:** Le SPTS-C avait une structure à un facteur qui expliquait 48 % de la variance et démontrait une bonne fiabilité ainsi qu’une bonne validité de la construction. Les scores obtenus au SPTS-C au départ prédisaient les niveaux d’interférence de la douleur au suivi mais pas l’intensité de la douleur ou le désagrément de la douleur.

**Conclusions:** Les résultats présentent des preuves préliminaires des propriétés psychométriques du SPTSC et le rôle potentiel de la sensibilité à la traumatisation de la douleur dans les résultats liés à la douleur chronique pédiatrique.

Pediatric chronic pain (i.e., pain lasting for 3 months or longer) affects 11% to 38% of youth,^1^ costs US$19 billion annually,^2^ is disabling for up to 5% of youth,^3^ and frequently persists into adulthood.^4^ Pediatric chronic pain often co-occurs with, follows the onset of, or precedes mental health symptoms (e.g., anxiety, depressive symptoms).^5^ Impacted youth can remain vulnerable to a later onset of mental health conditions even if their pain resolves.^6^ Pediatric chronic pain also shows substantial comorbidity with posttraumatic stress symptoms (i.e., intrusive thoughts/images, avoidance of trauma reminders, negative alterations in cognition/mood, and altered reactivity),^7^ yet existing research has mostly focused on anxiety^8^ and depressive^9^ symptoms in the context of pediatric chronic pain. Less attention has been given to posttraumatic stress symptoms, despite evidence that elevated posttraumatic stress symptoms often co-occur with pediatric chronic pain. Indeed, 32% of children seen in a tertiary-level chronic pain program reported clinically significant levels of posttraumatic stress symptoms compared to 8% of pain-free peers.^10^ Conceptual models posit that pediatric chronic pain and posttraumatic stress symptoms share mutually maintaining factors (e.g., avoidance, anxiety sensitivity).^7^ In a sample of youth with chronic pain, higher baseline levels of posttraumatic stress symptoms predicted higher levels of pain interference at follow-up, controlling for baseline pain interference.^11^ Thus, posttraumatic stress symptoms may be either a driving factor in the maintenance of, or share vulnerabilities with, pediatric chronic pain.

Sensitivity to pain traumatization is a novel construct that has been posited to underlie the co-occurrence of chronic pain and posttraumatic stress symptoms.^12^ The original construct was developed based on analysis of the hierarchical structure of pain-related anxiety constructs (i.e., pain catastrophizing, anxiety sensitivity, and pain anxiety) using a bifactor hierarchical model.^12,13^ Bifactor hierarchical models allow for the analysis of correlated measures with a significant degree of item overlap^13^ and identification of a general factor that is not qualitatively different from the examined measures. Instead, the general factor represents another possible source of item variance. Kleiman and colleagues’ analyses revealed a general factor that represented an individual’s likelihood to develop emotional, behavioral, somatic, and cognitive reactions in response to pain that resembled a traumatic stress reaction.^12^ Sensitivity to pain traumatization is different from the abovementioned pain-related anxiety constructs because it captures an individual’s propensity to react to pain in a way to that is similar to a reaction to trauma and goes beyond cognitive (i.e., pain catastrophizing) and affective (i.e., fear of somatic sensations associated with anxiety, fear of pain) reactions to pain.

Further, the general factor was strongly related to posttraumatic stress symptoms, but not depressive symptoms, which suggested trauma-like features of the new factor (i.e., sensitivity to pain traumatization).^14^ Of note, sensitivity to pain traumatization scores, but not posttraumatic stress symptom scores, predicted the development of persistent postsurgical pain in individuals who underwent major surgery, suggesting that sensitivity to pain traumatization is distinct from posttraumatic stress symptoms and may better predict the onset of persistent postsurgical pain in adults than posttraumatic stress symptoms.^12^ Based on these findings, a measure of sensitivity to pain traumatization (i.e., Sensitivity to Pain Traumatization Scale [SPTS-12]) was developed.^14^ The SPTS-12 demonstrated moderate to excellent validity and reliability in community and clinical (i.e., postsurgery) adult samples.^14^ The SPTS-12 has been recently adapted for parents of youth with chronic pain. The new measure (i.e., the Sensitivity to Pain Traumatization Scale–Parent version [SPTS-P]) assesses a parent’s propensity to develop a traumatic stress reaction in response to their child’s pain and demonstrated good reliability and moderate construct validity.^15^ Higher parent sensitivity to pain traumatization scores predicted higher levels of monitoring and protective parenting behaviors^15^ but not youth pain outcomes.

Sensitivity to pain traumatization may play a key role in the onset and maintenance of pediatric chronic pain; however, a measure directly assessing the construct in youth has not yet been developed. The current study aimed to fill this gap by creating a child version of the SPTS-12 (Sensitivity to Pain Traumatization Scale–Child version [SPTS-C]) and evaluating its psychometric properties in a sample of youth with chronic pain. Given the psychometric qualities of the original SPTS-12,^14^ we hypothesized that the SPTS-C would (1) have one factor; (2) be reliable (i.e., have at least adequate internal consistency [i.e., Cronbach’s alpha ≥ 0.070]^16^ and test–retest reliability; i.e., interclass correlation coefficient ≥ 0.40^17^); (3) have good convergent (i.e., be strongly correlated to a measure of posttraumatic stress symptoms), discriminant (i.e., have a significantly weaker relationship with a measure that is theoretically distinct from sensitivity to pain traumatization; e.g., a measure of attentional control), and concurrent (i.e., be strongly correlated with pain anxiety measures) validity; (4) demonstrate predictive validity (i.e., predict pain intensity, unpleasantness, and interference at 3-month follow-up).

## Materials and Methods

### Participants

Participants consisted of a sample of 175 youth with chronic pain (73% girls, *M*_age_ = 14.31; SD = 2.22) and one of their parents. Youth were recruited as part of the Pain and Mental Health study, which is a larger, longitudinal research program aimed at investigating underlying mechanisms implicated in co-occurring pediatric chronic pain and mental health symptoms. This study focused specifically on examining the factor structure and psychometric properties of a child version of the SPTS (SPTS-C). The aims of this study are thus distinct from previously published data from the Pain and Mental Health study.^11,18–25^

Youth were eligible for this study if they were between the ages of 10 and 18 years and were referred to a multidisciplinary chronic pain program for assessment and/or treatment. Participants were recruited from three outpatient clinics (Headache, Gastroenterology, and Complex Pain) located within the pain and rehabilitation center at Alberta Children’s Hospital. At the time of recruitment, youth were confirmed to have chronic pain that had been present for a period of 3 months or more. Exclusion criteria included the presence of serious physical, cognitive, or psychiatric conditions (e.g., cancer, psychotic disorder, developmental disorder, cognitive impairment) and/or an inability to understand/speak English.

All youth received treatment for their chronic pain at one of the outpatient clinics. The treatment typically included intake and monthly follow-up sessions with a pain specialist (i.e., a medical doctor or a nurse practitioner), medication, occupational therapy, physical therapy, and/or group/individual sessions with a pain psychologist. Treatment data were not collected to reduce the participants’ burden.

### Procedure

All study procedures were approved by the institution’s health research ethics board (REB15-3100). During the initial appointments at the abovementioned clinics, clinical staff obtained consent from families interested in participating in research studies to be contacted by the study team. Research staff also generated a list of families who were participating in a clinical outcomes study and who had consented to be contacted about future studies. Research staff contacted potential participants to provide information about the study and to invite them to participate. Interested youth and their parents were screened for eligibility over the phone, and verbal consent for study participation was obtained. Written informed consent was obtained through online consent forms that were emailed to participating families after the initial phone call. Youth below the age of 14 years were asked to provide their assent, and parents of these youth provided written informed consent for themselves and for their child to participate in the study.

Parents and youth each completed an online questionnaire via Research Electronic Data Capture (REDCap), a secure web-based data collection tool.^26^ Parents completed questionnaires that assessed sociodemographic information. Youth completed self-report measures to assess baseline levels of sensitivity to pain traumatization, posttraumatic stress symptoms, attentional control, and pain-related anxiety constructs (i.e., pain anxiety, anxiety sensitivity, pain catastrophizing), as well as baseline pain characteristics (e.g., location, duration, intensity, interference, unpleasantness). The Kiddie Schedule for Affective Disorders and Schizophrenia for School-Age Children–Present and Lifetime Version (K-SADS-PL), a semistructured diagnostic clinical interview, was administered to youth and their parents via telephone at baseline to assess for the presence of internalizing mental health disorders at the diagnostic level and to characterize the sample. All interviews were audio-recorded and were conducted by trained undergraduate honors students in psychology and graduate students in clinical or counseling psychology. Approximately 3 months after completing the baseline questionnaire, youth reported follow-up levels of sensitivity to pain traumatization as well as their pain intensity, unpleasantness, and interference. Youth and their parents each received an honorarium ($10 gift card for parents; $20 gift card for youth) at each testing point (at baseline and at 3-month follow-up).

### Measures

#### Child Sensitivity to Pain Traumatization

The SPTS-C was developed by J.K., M.N., and M.P. by rewording the original SPTS-12 items to make them age appropriate and easily understood by children aged 8 to 18 years. Specifically:

1. We identified phrases and words (e.g., “avoid activities,” “pain sensations”) from the SPTS-12 that might not be understood by, or clear to, children aged 8 to 18 years.
2. We cross-checked these and other words and phrases from the SPTS-12 that appear in other validated pediatric pain-related and trauma-related measures used with children aged 8 or older, including the Pain Catastrophizing Scale–Child Version (PCS-C), Fear of Pain Questionnaire (FOPQ), Child PTSD Symptom Scale–5 (CPSS-5), Clinician-Administered PTSD Scale for DSM-5–Child/Adolescent Version, the Pain Questionnaire, and the Child Pain Anxiety Symptoms Scale (CPASS). The following words and phrases appearing in SPTS-12 items are also used in the measures listed above and were adopted for the SPTS-C: (a) “When I am in pain” (multiple items; PCS-P); (b) “terrible” (item 4; PCS-P, FOPQ); (c) “I can’t stand” (item 7; PCS-P); (d) “reminds me of” (item 2; CPSS-5); (e) “avoid activities” (item 3; CPSS-5, FOPQ); (f) “pain bothers me” (item 5; Pain Questionnaire); (g) “things don’t feel real” (item 11; Clinician-Administered PTSD Scale for DSM-5–Child/Adolescent Version).
3. We identified the following words and phrases from the SPTS-12 that needed to be changed to accommodate a lower readability level: (a) “distant” (item 8) was changed to “far away”; (b) “medications” (item 9) was changed to “medicines”; (c) “pain sensations terrify me” was changed to “feelings of pain scare me” (item 10); (d) “pain seems to bother me” (item 5) was changed to “pain seems to bother and upset me.”
4. We opted to change the anchors of the SPTS-12’s 5-point (ranging from 0 to 4) Likert scale to *not at all true, a little true, between a little and a lot true, a lot true*, and *very much true*. These anchors are used in the pain affect question of the Pain Questionnaire^27^ and thus were deemed to be more easily understood by children and adolescents.

We conducted a focus group with 13 children aged 7 to 12 years (eight girls and five boys) to review the 12 items of the SPTS-C for readability and comprehension. First, children individually reviewed various wordings of the scale items, chose their preferred wording for each item, or suggested an alternative one. Then, the children and first and senior authors met as a group to discuss any confusing item wordings and to come to an agreement about the best wording for each item.

**Figure 1.**
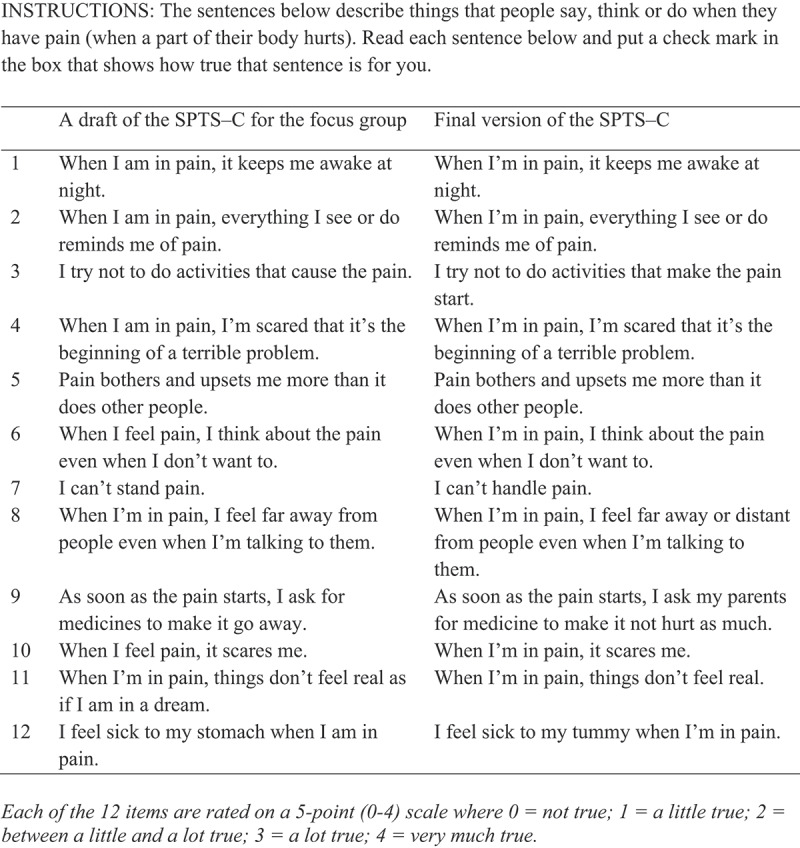
Sensitivity to Pain Traumatization Scale–Child version. Each of the 12 items are rated on a 5-point (0–4) scale where 0 =* not true*, 1 =* a little true*, 2 =* between a little and a lot true*, 3 =* a lot true*, 4 =* very much true*.

Based on children’s feedback, we made further changes to the measure (see Figure 1). Specifically, (1) “cause the pain” (item 3) was changed to “make the pain start”; (2) “when I feel pain” and “being in pain” stems were changed to “when I’m in pain” (multiple items); (3) “I can’t stand pain” (item 7) was changed to “I can’t handle pain”; (4) “distant” was added back to item 8; (5) item 9 was changed from “As soon as the pain starts, I ask for medicines to make it go away” to “As soon as the pain starts, I ask my parents for medicine to make it not hurt as much”; (6) item 11 was simplified by removing “I feel like I’m in a dream”; (7) “stomach” was replaced by “tummy” (item 12). The final scale (see Figure 1) had an average grade-level readability of Grade 1 (Flesh-Kincaid Grade Level = 1; Flesh Reading Ease = 100.5 [very easy to read]; Dale-Chall Readability = 0.6 [below first grade]) and was expected to be understood by children as young as 6 to 8 years of age.^28^

#### Posttraumatic Stress

##### Child PTSD Symptoms Scale (Convergent Validity)

Youth baseline posttraumatic stress symptoms were assessed using the CPSS-5, a 27-item scale that assesses posttraumatic stress disorder (PTSD) symptomatology based on *Diagnostic and Statistical Manual of Mental Disorders*, 5th edition (DSM-5) diagnostic criteria.^29,30^ Youth were asked to identify their “scary or upsetting” event and rate the frequency of 20 items (which correspond to PTSD symptom clusters in the DSM-5) on a 5-point Likert scale ranging from 0 (*not at all*) to 4 (*six or more times a week/almost always*). Seven additional items assess functional impairment and are rated as present or absent; these items are not included in the total CPSS-5 score. Higher total symptom severity scores (ranging from 0 to 80) are indicative of higher levels of PTSD symptoms and impairment. A score of 31 or greater indicates a probable PTSD diagnosis.^29^ The CPSS-5 has been used previously in research on youth with chronic pain^10^ and is based on the CPSS-4, which has shown excellent reliability and validity.^30,31^ In the current study, internal consistency of the CPSS-5 was excellent (*α* = 0.95).

##### Kiddie Schedule for Affective Disorders and Schizophrenia for School-Age Children–Present and Lifetime Version–Present and Lifetime Version

The K-SADS-PL is a semistructured diagnostic interview that assesses current and past episodes of psychopathology in youth aged 6 to 18 years based on the DSM-5 criteria. The K-SADS-PL consists of a diagnostic screening interview, diagnostic supplements (administered as appropriate, based on responses during the screening interview to further examine mental health symptoms), and a summary lifetime diagnoses checklist. Where discrepancies occurred between parent and youth report, clinical judgment and professional consultation were used to complete the summary lifetime diagnosis checklist. The K-SADS-PL has good reliability and predictive validity.^32^

To determine the presence or absence of posttraumatic stress disorder, trained researchers asked youth whether they had experienced one or more traumatic events (e.g., being exposed to domestic violence, witnessing a crime, being confronted with traumatic news) and assessed whether youth were experiencing one or more posttraumatic stress symptom(s) (e.g., flashbacks, avoidance of trauma reminders). If youth had experienced a traumatic event and/or endorsed at least one of six posttraumatic stress symptoms, a trauma-related disorders supplement was administered. The supplement includes 15 semistructured questions about posttraumatic stress symptoms and their impact on youth’s daily, academic, and social functioning. The obtained information was checked against the DSM-5 criteria to determine whether a diagnosis of PTSD was warranted. For the purposes of the present study, participants’ baseline PTSD status (i.e., probable, definitive, or not present) was used to characterize the sample.

#### Attentional Control Scale (Discriminant Validity)

At baseline, youth completed the Attentional Control Scale (ACS), which is a 20-item measure that assesses an individual’s reported level of attentional control.^33^ Items are rated on a 4-point Likert scale, with higher total scores indicating greater self-reported attentional control. The measure consists of two subscales that assess attention focusing and attention shifting. The ACS has demonstrated good reliability,^33^ as well as good construct validity and a reliable factor structure in pediatric samples.^34^ For the analyses reported in this study, the ACS total score was calculated excluding item 9, as recommended to enhance factor structure fit and increased measure precision.^34–37^ In the current study, internal consistency of the ACS was good (*α* = 0.83).

#### Pain-Related Anxiety Constructs^a^ (Concurrent Validity)

##### Child Pain Anxiety Symptoms Scale

At baseline, youth completed the CPASS, which is a modified version of the adult Pain Anxiety Symptoms Scale–Short Form adapted for use with children and adolescents.^38^ Youth are asked to indicate the extent to which each item describes them when they are in pain on a 6-point Likert scale with responses ranging from 0 (*means you never think, act, or feel that way*) to 5 (*means that you always think, act, or feel that way*). The CPASS has been shown to have a factor structure similar to the adult measure and has demonstrated strong internal consistency and good construct and discriminant validity in nonclinical and clinical samples.^38,39^ In the current study, internal consistency of the CPASS was excellent (*α* = 0.93).

##### Pain Catastrophizing Scale-Child Version

The PCS-C is a 13-item measure that was used to assess youth’s catastrophic thoughts and feelings about their pain at baseline.^40^ Each item is rated on a 5-point Likert scale ranging from 0 (*not at all*) to 4 (*extremely*), and youth are asked to indicate how strongly they endorse each statement when they are in pain. Items are summed to produce both a total score and three subscale scores (Magnification, Rumination, and Helplessness). The PCS-C has demonstrated good validity and reliability in community and clinical samples of youth with chronic pain.^40^ In the current study, internal consistency of the PCS-C was excellent (*α* = 0.94).

##### Childhood Anxiety Sensitivity Index

The Childhood Anxiety Sensitivity Index (CASI) is an 18-item self-report measure that assesses the tendency to interpret anxiety-related bodily sensations as threatening and was used to assess anxiety sensitivity in youth.^41^ At baseline, youth rated the extent to which each statement described them on a 3-point Likert scale ranging from 1 (*none*) to 3 (*a lot*), with higher scores indicating higher anxiety sensitivity. The CASI has been shown to have adequate test–retest reliability and high internal consistency in community and clinical samples of youth.^41^ In the current study, internal consistency of the CASI was excellent (*α* = 0.91).

#### Pain Outcomes (Predictive Validity)

Baseline and follow-up levels of pain intensity over the past 7 days was assessed using an 11-point numerical rating scale ranging from 0 (*no pain*) to 10 (*worst pain possible*). The numerical rating scale has been shown to be a valid and reliable measure for assessing pain intensity in youth with chronic pain.^42,43^ Pain unpleasantness was rated on a 5-point Likert scale assessing how much pain was bothersome over the past 7 days (ranging from 0 = *not at all* to 4 = *very much*).

The 4-item Pain Interference short form from the Patient-Reported Outcomes Measurement Information System (PROMIS-25) Pediatric Profile (v1.0) was used to assess baseline and follow-up pain interference in youth. The PROMIS instruments were developed by the National Institutes of Health using item response theory to assess a variety of physical and mental health symptoms across the life span.^44^ The four items of the Pain Interference subscale are rated using a 5-point Likert scale ranging from 0 (*never*) to 4 (*almost always*), with higher scores indicating greater interference over the past 7 days. This scale has demonstrated validity in youth with chronic pain.^45^ Total raw scores were transformed into standardized *T* scores for analysis in the current study.

#### Sample Characteristics

##### Sociodemographics

Parents provided sociodemographic information on their child’s age, gender, and race/ethnicity.

##### Additional Pain Characteristics (Locations, Frequency, Duration)

Youth reported their pain frequency, location(s), and duration at baseline. Participants reported the duration of their pain problem in years and months. Participants selected locations from a checklist of six body location options (e.g., stomach, head) to signify where they experienced the most aches or pains in the past 7 days.^46^ Pain frequency was assessed over the past 7 days using a 5-point Likert scale (ranging from 0 = *not at all* to 4 = *daily*).

### Statistical Analyses

Analyses were conducted using IBM SPSS (v27)^47^ and RStudio (v1.1.383).^48^ Descriptive statistics (i.e., means and standard deviations) were calculated to characterize continuous variables. Frequency statistics were used for categorical variables. *T* tests were conducted to examine differences in SPTS-C scores as a function of sociodemographic characteristics (i.e., gender and race/ethnicity). A Pearson’s correlation between SPTS-C scores and age was performed.

#### Exploratory Factor Analysis

Exploratory factor analysis (EFA) was conducted in RStudio using the polychoric correlation matrix and principal axis factoring estimation and the “psych,” “Hmisc,” and “poly_cor” packages in R to evaluate the scale items and factor structure of the SPTS-C. The number of factors to retain was determined through examination of the scree plot, parallel analysis, Velicer’s minimum average partial test, the root mean square residual, and the ratio of the first initial eigenvalue to the second initial eigenvalue. The Kaiser-Meyer-Olkin measure of sampling adequacy was used to determine whether data were suitable for EFA. Bartlett’s test of sphericity was performed to examine whether item correlations were sufficient to conduct EFA. The SPTS-C is a new measure based on the SPTS-12. Because the SPTS-C assessed a new construct (i.e., *child* SPT) in a novel clinical population (i.e., youth with chronic pain up to the age of 18 years), EFA, rather than confirmatory factor analysis, was conducted.^49^

#### Reliability Analyses

To examine the internal consistency of the SPTS-C, Cronbach’s alpha (*α*), corrected item-total correlations, and Cronbach’s *α*-if-item-deleted were calculated. To examine the measure’s test–retest reliability, Pearson’s correlations between the baseline and follow-up SPTS-C scores were performed and an interclass correlation estimate of baseline and follow-up SPTS-C mean scores was calculated based on a mean rating (*k* = 2), absolute agreement, two-way mixed-effects model.^50^

#### Validity Analyses

Pearson’s correlations were used to examine the construct validity of the SPTS-C. Specifically, to examine convergent validity, Pearson’s correlations were performed between the SPTS-C and CPSS-5 (i.e., measure of posttraumatic stress symptoms that is conceptually related, yet not specific, to pain). To examine discriminant validity, Pearson’s correlations were conducted between the SPTS-C and the ACS (i.e., measure of attentional control that is theoretically distinct from sensitivity to pain traumatization). Correlation coefficients of 0.30 or larger are considered large.^51^ Therefore, a correlation of *r* ≥ 0.30 between the sensitivity to pain traumatization measure and the measure of posttraumatic stress symptoms would imply adequate convergent validity, whereas a small to medium correlation (*r* < 0.30) between the sensitivity to pain traumatization measure and the measure of attentional control would indicate adequate discriminant validity. Additionally, discriminant validity would be achieved if the correlation between sensitivity to pain traumatization and posttraumatic stress symptoms scores was significantly greater than the correlation coefficient between sensitivity to pain traumatization and attentional control scores. The statistical significance of the coefficients’ difference was tested using a *Z* (normal curve) test.^52^

To examine the concurrent validity (i.e., associations between child sensitivity to pain traumatization and relevant pain-related anxiety constructs) of the SPTS-C, Pearson’s correlations between sensitivity to pain traumatization, anxiety sensitivity, pain anxiety, and pain catastrophizing scores were performed.

To examine the predictive validity of the sensitivity to pain traumatization measure, first, Pearson’s correlations between sensitivity to pain traumatization scores and youth pain outcomes (i.e., pain intensity, pain unpleasantness, and pain interference at follow-up) were conducted. Significant associations were followed up by hierarchical linear regression models that examined whether sensitivity to pain traumatization scores were predictive of follow-up pain characteristics, while controlling for corresponding baseline pain characteristics.

To further examine the unique contribution of sensitivity to pain traumatization to youth pain outcomes, hierarchical regression models that revealed sensitivity to pain traumatization to be a significant predictor of youth pain outcomes were followed up with dominance analyses.^53^ Given hypothesized strong correlations between sensitivity to pain traumatization and pain-related anxiety constructs (Hypothesis 3), any regression analyses that include both sensitivity to pain traumatization and the pain-related anxiety constructs would be vulnerable to multicollinearity. Even though the overall variance explained by a set of predictors is stable, coefficient estimates (i.e., beta weights) of strongly correlated predictors become sensitive to minor changes in the statistical models and can no longer be used to reliably compare relative contributions of individual predictors. Dominance analysis allows to account for strong correlations among individual predictors. As a part of dominance analyses, all possible regression models are conducted, and an average increase in criterion’s explained variance associated with each individual predictor is calculated.^53^ In the follow-up dominance analyses, we included sensitivity to pain traumatization scores along with pain- and anxiety-related constructs (i.e., pain catastrophizing, anxiety sensitivity, and pain anxiety) to predict pain outcomes, while controlling for corresponding baseline pain characteristics.

## Results

### Data Preparation

A total of 182 youth were recruited. Seven participants did not complete at least one of the SPTS-C items at baseline and were excluded from the final analyses.^2^ At 3-month follow-up, 26 youth did not complete at least one of the SPTS-C items and were excluded from the test–retest reliability analyses. For the remaining measures, total scores were prorated if youth completed at least 80% of items. Missing data ranged from 1% on the measure of attentional control (*n* = 2) to 5% on the measure of pain catastrophizing (*n* = 9). Little’s missing completely at random test^54^ was not significant, *χ*^2^(91) = 86.30, *P* = 0.62. Thus, data were assumed missing at random.

### Sample Characteristics

Baseline sociodemographic characteristics, pain, and past traumatic event characteristics obtained from the measure of posttraumatic stress symptoms are summarized in Table 1. Most youth were female (73%), white (77%), and, on average, 14.31 years old (SD = 2.22). Participants reported an average pain duration of 2.69 years (SD = 3.04; range = 3 months to 12 years). Most youth reported having headaches (72%), pain in muscles and joints (24%), and other pain (25%). At baseline, 46% of youth reported having pain on a daily basis. On average, baseline pain intensity was 5.51/10 (SD = 1.83). At 3-month follow-up, average pain intensity was 5.33/10 (SD = 2.05). Youth reported 2.01/4 (SD = 0.90) baseline and 1.64/4 (*SD* = 0.98) follow-up average levels of pain unpleasantness. Average level of pain interference was 55.04 (SD = 9.24) at baseline and 52.79 (SD = 9.59) at follow-up. Seventeen percent of youth reported clinically elevated levels of posttraumatic stress symptoms (*n* = 30), and 11% of participants met criteria for probable or definite PTSD over the lifetime (*n* = 18), as assessed by the K-SADS clinical interview.

**Table 1.** Baseline sociodemographic, pain, and past traumatic characteristics of the sample, *N* = 175.

Characteristic	
Age, *M* (SD) in years, range 10–18	14.31 (2.22)
Gender, *n* (%)	
Female	127 (73)
Male	48 (27)
Race/ethnicity, *n* (%)	
White	134 (77)
Bi- or multiracial	14 (8)
Other or do not want to answer	13 (7)
Arab/West Asian	3 (2)
Black/African American	3 (2)
South Asian	3 (2)
Latin American	2 (1)
Indigenous	2 (1)
Filipino	1 (<1)
Pain duration, *M* (SD) in years, range 0.25–12	2.69 (3.04)
Pain frequency, *n* (%)	
Not at all	5 (3)
Once per week	13 (7)
2 to 3 times per week	50 (29)
4 to 6 times per week	26 (15)
Daily	81 (46)
Pain locations, *n* (%)^a^	
Head	126 (72)
Other	43 (25)
Muscle and joints	42 (24)
Stomach	32 (18)
Legs	25 (14)
Chest	19 (11)
Traumatic event, *n* (%)	
Not applicable (child recorded N/A or left blank)	49 (28)
Other (e.g., death of a pet, natural disaster)	27 (15)
Death	21 (12)
Physical illness or hospitalization	15 (9)
Multiple traumatic events	12 (7)
Mental illness or addiction	9 (5)
Chronic pain problem or pain-related experience (e.g., concussion)	8 (4)
Physical, verbal, or sexual abuse	8 (4)
Parents’ divorce or family-related conflict	8 (4)
Car accident	6 (3)
Social difficulties	4 (2)
Academic difficulties	4 (2)
Suicide-related trauma, self-harm	4 (2)

^a^Percentages add up to greater than 100 because 40% of youth endorsed multiple pain locations.

### Factor Analysis

The Kaiser-Meyer-Olkin measure of sampling adequacy was 0.89, indicating that the present data were suitable for EFA. Bartlett’s test of sphericity was significant, *χ*^2^(66) = 736.33, *P* < 0.001, which indicated sufficient item correlations to conduct EFA. All items loaded onto one factor at a value of 0.40 or higher (except for item 9; see Table 2 for factor loadings). An examination of the scree plot suggested that a one-factor solution was appropriate. Results from a parallel analysis using real-data eigenvalues and Velicer’s minimum average partial test yielded a two-factor solution (see Supplemental Materials, Table S1 for factor loadings). Given the divergent results, a final maximum method agreement procedure was conducted using the n_factors function from the nFactors package. The n_factors function runs multiple existing procedures to determine how many factors to retain from a factor analysis and returns the number of factors to retain based on the maximum consensus between methods. The n_factors function analysis determined that the maximum consensus between methods was to retain one factor, which was supported by 22.22% of the methods tested. Thus, based on the scree plot and results from the maximum method agreement procedure, a one-factor solution was retained. The root mean square residual was 0.088, indicating a good fit and providing support for the one-factor model. In addition, the ratio of the first (5.82) to the second (0.66) eigenvalue was >4. The one-factor model accounted for 48.5% of the variance.

**Table 2.** Item-total statistics and factor loadings for the one-factor solution of the SPTS-C, *N* = 175.

SPTS-C item	*M* (SD)	*α*-if-item-deleted	Inter-item total *r*	Factor loading	Communality
1. When I’m in pain, it keeps me awake at night.	1.57 (1.18)	0.879	0.441	0.677	0.458
2. When I’m in pain, everything I see or do reminds me of pain.	0.64 (0.95)	0.864	0.731	0.684	0.467
3. I try not to do activities that make the pain start.	1.75 (1.35)	0.876	0.509	0.474	0.225
4. When I’m in pain, I’m scared that it’s the beginning of a terrible problem.	0.71 (1.10)	0.866	0.672	0.660	0.436
5. Pain bothers and upsets me more than it does other people.	1.06 (1.30)	0.864	0.680	0.669	0.448
6. When I’m in pain, I think about the pain even when I don’t want to.	1.43 (1.30)	0.863	0.707	0.763	0.582
7. I can’t handle pain.	0.98 (1.19)	0.874	0.519	0.555	0.308
8. When I’m in pain, I feel far away or distant from people even when I’m talking to them.	1.06 (1.24)	0.872	0.563	0.653	0.427
9. As soon as the pain starts, I ask my parents for medicine to make it not hurt as much.	1.03 (1.22)	0.884	0.364	0.302	0.091
10. When I’m in pain, it scares me.	0.64 (1.05)	0.865	0.700	0.641	0.411
11. When I’m in pain, things don’t feel real.	0.85 (1.14)	0.866	0.667	0.569	0.324
12. I feel sick to my tummy when I’m in pain.	1.05 (1.23)	0.877	0.474	0.652	0.425
Total SPTS-C score at baseline (*n* = 175), range 0–48, interquartile range = 11.5, median = 11, skewness = 1.25 (SE = 0.20), kurtosis = 1.98 (SE = 0.29)	12.76 (9.41)	—	—	—	—
Total SPTS-C score at follow-up (*n* = 149), range 0–48, interquartile range = 11, median = 7, skewness = 1.31 (SE = 0.20), kurtosis = 1.62 (SE = 0.29)	9.50 (8.65)	—	—	—	—

### Reliability

#### Internal Consistency

The SPTS-C demonstrated good internal consistency (*α* = 0.88). Cronbach’s *α*-if-item-deleted analyses demonstrated that SPTS-C internal consistency remained the same if any of the items were deleted (*α *= 0.86–0.88; Table 2). Corrected item-total correlations ranged from 0.36 to 0.73.

#### Test-Retest Reliability

The SPTS-C baseline and follow-up scores were significantly correlated, *r* = 0.58, *P* < 0.001. The intraclass correlation coefficient was 0.41, *P* = 0.015.

### Construct Validity

#### Convergent Validity

The baseline SPTS-C scores were significantly positively correlated with the baseline scores of posttraumatic stress symptoms, *r* = 0.54, *P* < 0.001 (Table 3). The coefficient of determination (*r*^2^) showed that 29.16% of variance was shared between the measures of sensitivity to pain traumatization and posttraumatic stress symptoms. Further, youth who had clinically elevated levels of posttraumatic stress symptoms (i.e., scored above the clinical cutoff on the associated measure) had significantly higher sensitivity to pain traumatization scores (*M* = 22.10, SD = 11.66) than youth who scored below the clinical cutoff (*M* = 10.76, SD = 7.62), *t*(34.66) = 5.09, *P* < 0.001. Similarly, youth who met criteria for posttraumatic stress disorder, as assessed by the K-SADS, had significantly higher sensitivity to pain traumatization scores (*M* = 20.67, SD = 10.95) compared to youth who did not met criteria for PTSD (*M* = 11.71, SD = 8.64), *t*(165) = 4.03, *P* < 0.001.

**Table 3.** Means, standard deviations, and Pearson’s correlations between the SPTS-C and related constructs.

Variable	2	3	4	5	6	7	8	9	10	11	12	13	*M* (SD)
1. Baseline SPTS-C	0.58***	0.54***	−0.27***	0.64***	0.84***	0.82***	0.25**	0.48***	0.40***	0.23**	0.34***	0.42***	12.76 (9.41)
2. Three-month SPTS-C	—	0.38***	−0.20*	0.48***	0.53***	0.53***	0.13	0.26**	0.34***	0.28***	0.40***	0.51***	9.50 (8.65)
3. Baseline CPSS-5		—	−0.43**	0.63***	0.54***	0.52***	0.31***	0.41***	0.39***	0.24**	0.30***	0.36***	16.76 (16.95)
4. Baseline ACS			—	−0.36***	−0.34***	−0.32***	−0.12	−0.33***	−0.27***	−0.08	−0.24**	−0.26**	49.47 (8.33)
5. Baseline CASI				—	0.69***	0.68***	0.23**	0.42***	0.46***	0.21*	0.32***	0.45***	27.79 (7.41)
6. Baseline CPASS					—	0.79***	0.34***	0.51***	0.44***	0.26**	0.34***	0.37***	32.30 (20.02)
7. Baseline PCS-C						—	0.32***	0.55***	0.45***	0.21*	0.29***	0.40***	18.61 (12.01)
8. Baseline pain intensity							—	0.61***	0.48***	0.56***	0.29***	0.25**	5.51 (1.83)
9. Baseline pain unpleasantness								—	0.52***	0.33***	0.44***	0.33***	2.01 (0.90)
10. Baseline pain interference									—	0.26**	0.28***	0.51***	55.03 (9.24)
11. Three-month pain intensity										—	0.58***	0.50***	5.33 (2.05)
12. Three-month pain unpleasantness											—	0.56***	1.64 (0.98)
13. Three-month pain interference												—	52.79 (9.59)

*P < 0.05. **P < 0.01. ***P < 0.001.

#### Discriminant Validity

The baseline SPTS-C scores were significantly negatively correlated with the baseline scores of attentional control (*r* = −0.27, *P* < 0.001; Table 3). The coefficient of determination (*r*^2^) demonstrated that 7.29% of variance was shared between the measures of sensitivity to pain traumatization and attentional control. The magnitude of the correlation between the measures of sensitivity to pain traumatization and posttraumatic stress symptoms was significantly greater than the correlation between the measures of sensitivity to pain traumatization and attentional control (*Z* = 6.18, *P* < 0.001).^52^

### Criterion Validity

#### Concurrent Validity

The baseline SPTS-C scores were significantly positively correlated with baseline scores of anxiety sensitivity (*r* = 0.64, *P* < 0.001), pain anxiety (*r* = 0.84, *P* < 0.001), and pain catastrophizing (*r* = 0.82, *P* < 0.001) scores (Table 3).

#### Predictive Validity

Baseline scores on the SPTS-C were significantly correlated with follow-up levels of pain intensity (*r* = 0.23, *P* = 0.004), pain unpleasantness (*r* = 0.34, *P* < 0.001), and pain interference (*r* = 0.42, *P* < 0.001). Based on these correlational analyses, three hierarchical linear regression models were conducted to examine whether sensitivity to pain traumatization predicts youth pain outcomes, while taking into account baseline levels of pain characteristics and key sociodemographic variables (i.e., age and gender; Table 4).

**Table 4.** Baseline SPTS-C scores and 3-month pain outcomes: hierarchical regression models.

Criterion variable at 3-month follow-up	Step	Predictor	*β*	Δ*R*^2^	Cumulative *R*^2^
Pain intensity (*n* = 148)	1	Youth sex	−0.10	0.28***	0.28***
		Youth age	0.02		
		Baseline pain intensity	0.51***		
	2	Baseline SPTS-C	0.12	0.01	0.29***
Pain unpleasantness (*n* = 145)	1	Youth sex	−0.16	0.23***	0.23***
		Youth age	0.01		
		Baseline pain unpleasantness	0.43***		
	2	Baseline SPTS-C	0.14	0.02	0.24***
Pain interference (*n* = 141)	1	Youth sex	−0.12	0.26***	0.26***
		Youth age	0.07		
		Baseline pain interference	0.46***		
	2	Baseline SPTS-C	0.25**	0.05**	0.31***

We used developmentally appropriate versions of the measures that were used in the original SPT papers^12,14^ to assess pain-related anxiety constructs. When data from the seven participants with one or more unanswered SPTS-C item(s) were included in the analyses, the pattern of findings remained unchanged.

*p < 0.05. **p < 0.01. ***p < 0.001.

Additionally, the baseline SPTS-C scores were significantly positively correlated with baseline levels of pain intensity (*r* = 0.25, *P* = 0.001), pain unpleasantness (*r* = 0.48, *P* < 0.001), and pain interference (*r* = 0.40, *P* < 0.001; Table 3). The coefficients of determination (*r*^2^) showed that the measure of sensitivity to pain traumatization shared 6.25% of variance with baseline pain intensity, 23.04% of variance with baseline pain unpleasantness, and 16% of variance with baseline pain interference.

##### Hierarchical Regression Model 1: pain Intensity

Youth gender, age, and baseline levels of pain intensity accounted for 28% of variance in follow-up levels of pain intensity, *F*(3, 144) = 18.69, *P* < 0.001. Above and beyond these covariates, baseline sensitivity to pain traumatization scores did not account for a significant amount of variance and was not a significant predictor of pain intensity at follow-up (*P* > 0.05). The overall model accounted for 29% of variance, *F*(4, 143) = 14.85, *P* < 0.001.

##### Hierarchical Regression Model 2: Pain Unpleasantness

Youth gender, age, and baseline levels of pain unpleasantness accounted for 23% of variance in follow-up levels of pain unpleasantness, *F*(3, 141) = 13.67, *P* < 0.001. Above and beyond these covariates, baseline sensitivity to pain traumatization scores did not account for a significant amount of variance and were not a significant predictor of pain unpleasantness at follow-up (*P* > 0.05). The overall model accounted for 24% of variance, *F*(4, 140) = 11.08, *P* < 0.001.

##### Hierarchical Regression Model 3: Pain Interference

Youth gender, age, and baseline levels of pain interference accounted for 26% of variance in follow-up levels of pain interference, *F*(3, 137) = 16.09, *P* < 0.001. Above and beyond these covariates, baseline sensitivity to pain traumatization scores accounted for 5% of variance in follow-up levels of pain interference, Δ*F*(1, 136) = 9.55, *P* = 0.002. The SPTS-C standardized beta weight indicated that higher levels of sensitivity to pain traumatization at baseline (*β* = 0.25, *P* = 0.002) predicted higher levels of pain interference at follow-up. The overall model accounted for 31% of variance, *F*(4, 136) = 15.21, *P* < 0.001.

###### Dominance Analyses for Model 3

To examine distinct contributions of baseline sensitivity to pain traumatization and pain- and anxiety-related constructs (i.e., anxiety sensitivity, pain catastrophizing, and pain anxiety) to pain interference at follow-up (controlling for baseline pain interference, youth gender, and youth age), we conducted dominance analyses.^53^ The overall model accounted for 33% of variance, *F*(7, 133) = 9.17, *P* < 0.001. In total, baseline sensitivity to pain traumatization, anxiety sensitivity, pain catastrophizing, and pain anxiety accounted for 6.5% of variance in follow-up levels of pain interference, above and beyond youth age, gender, and baseline pain interference, *F*(4, 133) = 3.20, *P* = 0.015. Of the 6.5%, anxiety sensitivity accounted for 38% (7.5% of total variance), baseline sensitivity to pain traumatization accounted for 32% (6% of total variance), pain catastrophizing accounted for 15% (3% of total variance), and pain anxiety accounted for 14% (3% of total variance).

### Relationships with Sociodemographic Variables

SPTS-C baseline scores were not significantly correlated with youth age (*r* = 0.08, *P* > 0.05). SPTS-C baseline scores for boys (*M* = 12.41, SD = 10.64) and girls (*M* = 12.89, SD = 8.94) did not significantly differ, *t*(173) = 0.30, *P* > 0.05. Similarly, SPTS-C scores for white (*M* = 12.31, SD = 9.29) and non-white (*M* = 14.41, SD = 9.77) participants did not significantly differ, *t*(173) = −1.20, *P* > 0.05.

## Discussion

The present study adapted a measure of sensitivity to pain traumatization, defined as a vulnerability to develop cognitive, affective, and behavioral reactions to pain that resemble a traumatic stress reaction, to be used with children (i.e., SPTS-C) and examined psychometric properties of the measure in a cohort of youth with chronic pain. An exploratory factor analysis of the SPTS-C yielded a one-factor solution. The measure demonstrated good internal consistency and test–retest reliability. Evidence for construct validity (i.e., convergent and discriminant) was limited. As hypothesized, baseline SPTS-C scores predicted pain interference at follow-up. Contrary to our hypotheses, SPTS-C scores did not predict follow-up levels of pain intensity or pain unpleasantness. Overall, the study provided preliminary evidence for the reliability and validity of the SPTS-C and warrants further examination of the scale in community and other clinical (e.g., postsurgical pain, acute injury, burns) pediatric samples.

Exploratory factor analysis demonstrated that the SPTS-C items loaded onto a single factor that accounted for 48.5% of variance and fit the data well. This is consistent with the single-factor structure of the original sensitivity to pain traumatization measure that accounted for 46.1% (community sample of undergraduate students) to 52.2% (adults with chronic postsurgical pain) of variance.^14^ This is also consistent with a parent version of the measure that accounted for 44.9% of variance in a sample of parents of youth with chronic pain.^15^ As hypothesized, the SPTS-C demonstrated good internal consistency and test–retest reliability. These findings provide preliminary evidence that the SPTS-C is reliable for use in clinical pediatric chronic pain samples with a variety of chronic pain conditions (e.g., headaches, complex pain). Of note, item 9 (i.e., “As soon as the pain starts, I ask my parents for medicine to make it not hurt as much”) had the lowest factor loading, similar to the corresponding item in the original measure and the parent version.^14,15^ Given that the present study is a preliminary investigation of the new scale, the item was retained, and the wording was not changed. Future examinations of the scale may consider either removing the item or rewording it.

The evidence for construct validity of the SPTS-C was limited. The SPTS-C had a strong correlation with a measure of posttraumatic stress symptoms (a similar, but distinct, construct). The correlation between the SPTS-C and measure of posttraumatic stress symptoms was significantly greater than the correlation between the SPTS-C and a measure of attentional control (a distinct construct), which provide preliminary evidence for convergent and discriminant validity, respectively. Of note, youth who scored above the clinical cutoff on the self-report measure of posttraumatic stress symptoms and/or who met diagnostic criteria for PTSD as assessed by the clinical interview reported significantly higher levels of sensitivity to pain traumatization compared to youth whose posttraumatic stress symptoms scores were below clinical cutoff and/or did not meet diagnostic criteria. The latter may be interpreted as evidence for the measure’s known-group validity. Known-group validity reflects whether a measure can differentiate between members of two or more known groups (in this case, youth with and without clinically elevated posttraumatic stress symptoms).^55^ These findings provide support for the mutual maintenance model of co-occurring posttraumatic stress symptoms and pediatric chronic pain, which asserts that posttraumatic stress symptoms and pediatric chronic pain co-occur and mutually maintain each other through shared symptoms and vulnerability factors.^7^ Given the demonstrated associations between posttraumatic stress symptoms and sensitivity to pain traumatization and the finding that SPTS-C scores predicted pain interference scores 3 months later, sensitivity to pain traumatization may operate as a shared vulnerability factor.^12^

Posttraumatic stress symptomatology and its role in pediatric chronic pain is one of the novel research avenues that may enhance our understanding of pediatric chronic pain. The body of research examining posttraumatic stress symptoms co-occurring with pediatric chronic pain is growing. Posttraumatic stress symptoms are more prevalent in youth with, versus without, chronic pain.^10^ Higher baseline levels of posttraumatic stress symptoms predicted increases in pain interference over time in pediatric samples with chronic pain^11^ and traumatic brain injury^56^ but not vice versa. These findings suggest that posttraumatic stress symptoms play a key role in the maintenance of pediatric chronic pain over time. Sensitivity to pain traumatization may underlie the association between chronic pain and posttraumatic stress symptoms. Higher levels of sensitivity to pain traumatization imply a greater propensity, or vulnerability, to develop cognitive, affective, and behavioral reactions to pain resembling a traumatic stress reaction. In the context of chronic pain, pain flares may trigger these responses. This vulnerability may also maintain and elevate any co-occurring posttraumatic stress symptoms that are not necessarily pain related.

As expected, SPTS-C scores were significantly associated with pain-related anxiety constructs (i.e., anxiety sensitivity, pain anxiety, and pain catastrophizing). The magnitudes of correlations were comparable to the correlations demonstrated in adult samples with ongoing and chronic postsurgical pain.^12,14^ Dominance analyses demonstrated that sensitivity to pain traumatization and anxiety sensitivity predicted a larger proportion of variance in pain interference at follow-up compared to pain catastrophizing and pain anxiety. Taken together, these findings provide preliminary evidence that sensitivity to pain traumatization is similar, yet distinct, from other (possibly lower order) pain-related anxiety constructs, because it may be a higher order factor that provides another possible source of item variance.^12^

SPTS-C baseline scores were significantly correlated with baseline and follow-up levels of youth pain intensity, unpleasantness, and interference. When controlling for key demographic variables and baseline pain characteristics, SPTS-C baseline scores significantly predicted follow-up levels of pain interference but not follow-up levels of pain intensity or pain unpleasantness; thus, hypotheses regarding the predictive validity of the SPTS-C were partially supported. Sensitivity to pain traumatization is a propensity to develop traumatic stress–like reactions to pain (e.g., being hypervigilant, avoidant of anything that may bring on pain). These reactions may have a greater impact on, and predict, how much pain impacts daily functioning (i.e., pain interference) as opposed to predicting more subjective aspects of pain (i.e., intensity and unpleasantness). Moreover, though pain intensity and unpleasantness are key outcomes in pediatric pain rehabilitation,^57^ it is meaningful engagement in activities of daily living that have been prioritized as a treatment target by youth with chronic pain.^58^ Pain interference (i.e., the tendency for pain to hinder one’s participation in activities of daily living) is therefore a clinically relevant outcome precisely because it determines whether, and the extent to which, youth engage with the world around them. The present results show that sensitivity to pain traumatization is a risk factor for increased 3-month pain interference. It remains to be determined whether sensitivity to pain traumatization is a causal risk factor for pain interference.

Although further examinations the SPTS-C are needed, the preliminary evidence for the reliability and validity of the SPTS-C may be important to consider in clinical contexts. Individuals with heightened vulnerability to develop a traumatic stress–like reaction in response to pain may benefit from targeted interventions. Psychological factors have been shown to impact treatment response in children with chronic pain. For example, clinically significant levels of anxiety at the start of cognitive–behavioral therapy predicted poorer treatment response in a sample of children with chronic pain.^59^ Pediatric chronic pain treatments often target internalizing mental health symptoms (i.e., anxiety and depressive symptoms).^60^ Yet, past trauma and its impact on youth pain and health trajectories has rarely been assessed and addressed in clinical practice, despite its clinical relevance for treatment. For example, presence of PTSD at baseline in youth with functional abdominal pain was related to higher levels of anxiety and somatization after a brief cognitive–behavioral therapy intervention compared to youth without PTSD.^61^ In adult samples, innovative treatments that target posttraumatic stress symptoms and pain led to improved physical and mental health functioning.^62^ Therefore, identifying and addressing additional psychological pain–specific treatment targets that contribute to co-occurring mental health symptoms (i.e., trauma-related symptomatology, sensitivity to pain traumatization) may enhance existing interventions for pediatric chronic pain.

The study’s findings are limited by a relatively small sample size. Yet, it has been argued that samples with as few as 50 participants may be appropriate for exploratory factor analyses.^63^ Further, the one-factor solution of the SPTS-C is similar to the previously validated measures of sensitivity to pain traumatization for adults (i.e., SPTS-12^14^) and parents (i.e., SPTS-P^15^). Details of the treatment participants received for their chronic pain were not available. Received treatment might have changed the follow-up levels of sensitivity to pain traumatization, as well as the follow-up levels of pain intensity, unpleasantness, and/or interference, which might have impacted the SPTS-C’s test–retest reliability and predictive validity. Future investigations of the measure should analyze the effects of treatment on sensitivity to pain traumatization and its associations with pain. A proportion of enrolled participants were participants in other research projects. Youth participating in multiple research projects may have lower levels of pain and/or mental health symptoms, which, in turn, might have impacted the results. Future studies should control for multiple studies participation status. The evidence for the SPTS-C’s discriminant validity is limited because only one measure (i.e., attentional control) was used to examine it. Future studies should include additional measures that assess theoretically different constructs (e.g., conscientiousness, agreeableness). The present sample included only youth presenting with chronic pain at tertiary pain clinics. Future studies should examine the measure’s psychometric properties in larger primary care and community pediatric samples, as well as in pediatric samples at risk for experiencing a transition from acute to chronic pain (e.g., first-onset pain complaints in primary care, etc.) and those with traumatic injuries (e.g., traumatic brain injuries, pediatric burns). Predictive validity of the SPTS-C may be further examined in prospective cohorts of youth undergoing planned surgeries, which would allow for an investigation of whether child sensitivity to pain traumatization levels predict future levels of postsurgical pain and recovery trajectories. A longer-term follow-up assessment (e.g., 1 year) will allow further investigations of the stability of the SPTS-C over time. Investigation of the SPTS-C in the context of intervention studies for pediatric chronic pain will help to elucidate the SPTS-C’s sensitivity and responsiveness to change and the potential role of sensitivity to pain traumatization in treatment outcomes. Finally, the sample was predominantly white, which limits generalizability of findings. Both chronic pain and trauma are more prevalent in people with a racialized identity or lower socioeconomic status.^1,64–67^ The levels of sensitivity to pain traumatization, and its association with pain, may differ in youth exposed to more adversity. The SPTS-C should be investigated in more diverse samples to gain a better understanding of the construct and its relevance to youth of diverse backgrounds.

In conclusion, the present study developed a measure assessing sensitivity to pain traumatization in children. The SPTS-C demonstrated a one-factor structure and good reliability in a sample of youth with chronic pain; the evidence for the construct validity was limited. Future investigations of the measure’s psychometric qualities using community and other clinical samples (e.g., children undergoing major surgeries) are warranted.

## Supplementary Material

Supplemental Material

## Note

[a]. We used developmentally appropriate versions of the measures that were used in the original SPT papers^12,14^ to assess pain-related anxiety constructs.
