# Supplementary material for: The Sensitivity to Pain Traumatization Scale–Child Version (SPTS-C): Development and preliminary validation
Source: Can J Pain. 2024 Jan 12;8(1):2298769. doi: 10.1080/24740527.2023.2298769 (PMC10939150; doi:10.1080/24740527.2023.2298769)
Supplement: Supplemental Material [file UCJP_A_2298769_SM9725.docx]

*Table S1.* Item-total statistics and factor loadings for the two-factor solution of the Sensitivity to Pain Traumatization Scale–Child Version (SPTS–C), *N* = 175.

| SPTS-P item | Factor 1 loading | Factor 2 loading |
| --- | --- | --- |
| 1. When I’m in pain, it keeps me awake at night. | .682 | .237 |
| 1. When I’m in pain, everything I see or do reminds me of pain. | .682 | .110 |
| 1. I try not to do activities that make the pain start. | .473 | .132 |
| 1. When I’m in pain, I’m scared that it's the beginning of a terrible problem. | .656 | .068 |
| 1. Pain bothers and upsets me more than it does other people. | .670 | -.157 |
| 1. When I’m in pain, I think about the pain even when I don’t want to. | .761 | .112 |
| 1. I can’t handle pain. | .558 | .222 |
| 1. When I’m in pain, I feel far away or distant from people even when I’m talking to them. | .681 | -.415 |
| 1. As soon as the pain starts, I ask my parents for medicine to make it not hurt as much. | .303 | .158 |
| 1. When I’m in pain, it scares me. | .638 | .078 |
| 1. When I’m in pain, things don’t feel real. | .580 | -.297 |
| 1. I feel sick to my tummy when I’m in pain. | .652 | -.139 |
